# Allergic Reactions to Current Available COVID-19 Vaccinations: Pathophysiology, Causality, and Therapeutic Considerations

**DOI:** 10.3390/vaccines9030221

**Published:** 2021-03-05

**Authors:** Nicholas G. Kounis, Ioanna Koniari, Cesare de Gregorio, Dimitris Velissaris, Konstantinos Petalas, Aikaterini Brinia, Stelios F. Assimakopoulos, Christos Gogos, Sophia N. Kouni, George N. Kounis, GianFranco Calogiuri, Ming-Yow Hung

**Affiliations:** 1 Department of Internal Medicine, Division of Cardiology, University of Patras Medical School, 26221 Patras, Greece; 2 Department of Internal Medicine, Division of Cardiology, University Hospital of South Manchester NHS Foundation Trust, Manchester M23 9LT, UK; iokoniari@yahoo.gr; 3 Department of Clinical and Experimental Medicine, University of Messina Medical School, 98122 Messina, Italy; cesare.degregorio@unime.it; 4 Department of Internal Medicine, University of Patras, 26500 Patras, Greece; dimitrisvelissaris@yahoo.com; 5 Department of Allergology, 251 General Air Force Hospital, 11525 Athens, Greece; kpetalas@hotmail.com; 6 Allergy Practice, 26221 Patras, Greece; kbrinia@yahoo.gr; 7 Department of Internal Medicine, Division of Infectious Diseases, University of Patras Medical School, 26500 Patras, Greece; sassim@upatras.gr; 8 Covid-19 Unit, Papageorgiou General Hospital, 56403 Thessaloniki, Greece; gogos-grivas@hotmail.com; 9 Speech Therapy Practice, 26221 Patras, Greece; snkouni@yahoo.gr; 10 Department of Ophthalmology, “Saint Andrews” State General Hospital, 26335 Patras, Greece; gnkounis@gmail.com; 11 Pneumonology Department, Civil Hospital “NinettoMelli”, Pietro Vernoti, 72027 Brindisi, Italy; gf.calogiuri@libero.it; 12 Department of Internal Medicine, Immunology and Infectious Diseases, Section of Allergology and Clinical Immunology, University of Bari Medical School, 70121 Bari, Italy; 13 Department of Internal Medicine, Division of Cardiology, Shuang Ho Hospital, Taipei Medical University, New Taipei City 23561, Taiwan; myhung6@ms77.hinet.net; 14 Department of Internal Medicine, Division of Cardiology, School of Medicine, College of Medicine, Taipei Medical University, Taipei 110, Taiwan; 15 Taipei Heart Institute, Taipei Medical University, Taipei 110, Taiwan

**Keywords:** allergy, anaphylaxis, COVID-19, Kounis syndrome, vaccines

## Abstract

Vaccines constitute the most effective medications in public health as they control and prevent the spread of infectious diseases and reduce mortality. Similar to other medications, allergic reactions can occur during vaccination. While most reactions are neither frequent nor serious, anaphylactic reactions are potentially life-threatening allergic reactions that are encountered rarely, but can cause serious complications. The allergic responses caused by vaccines can stem from activation of mast cells via Fcε receptor-1 type I reaction, mediated by the interaction between immunoglobulin E (IgE) antibodies against a particular vaccine, and occur within minutes or up to four hours. The type IV allergic reactions initiate 48 h after vaccination and demonstrate their peak between 72 and 96 h. Non-IgE-mediated mast cell degranulation via activation of the complement system and via activation of the Mas-related G protein-coupled receptor X2 can also induce allergic reactions. Reactions are more often caused by inert substances, called excipients, which are added to vaccines to improve stability and absorption, increase solubility, influence palatability, or create a distinctive appearance, and not by the active vaccine itself. Polyethylene glycol, also known as macrogol, in the currently available Pfizer-BioNTech and Moderna COVID-19 mRNA vaccines, and polysorbate 80, also known as Tween 80, in AstraZeneca and Johnson & Johnson COVID-19 vaccines, are excipients mostly incriminated for allergic reactions. This review will summarize the current state of knowledge of immediate and delayed allergic reactions in the currently available vaccines against COVID-19, together with the general and specific therapeutic considerations. These considerations include: The incidence of allergic reactions and deaths under investigation with the available vaccines, application of vaccination in patients with mast cell disease, patients who developed an allergy during the first dose, vasovagal symptoms masquerading as allergic reactions, the COVID-19 vaccination in pregnancy, deaths associated with COVID-19 vaccination, and questions arising in managing of this current ordeal. Careful vaccine-safety surveillance over time, in conjunction with the elucidation of mechanisms of adverse events across different COVID-19 vaccine platforms, will contribute to the development of a safe vaccine strategy. Allergists’ expertise in proper diagnosis and treatment of allergic reactions is vital for the screening of high-risk individuals.

## 1. Introduction

Vaccines constitute the most effective medications in public health as they control and prevent the spread of infectious diseases. From the mid of the 20th century, many infectious diseases and their sequels have been effectively reduced, eliminated, and avoided through the use of vaccinations [1].

However, similar to other medications, allergic reactions can occur during vaccination. Vaccine-associated allergic reactions are neither frequent nor serious. However, anaphylactic reactions are potentially life-threatening, and can occur immediately, usually within minutes, after exposure to a vaccine. Such reactions are encountered rarely, but can cause serious complications. Following an allergic reaction after vaccination, it is difficult to discriminate and ascertain whether the reaction was caused by the vaccine itself or by other factors. The increased incidence of viral epidemics has rendered an increased use of various vaccines, and therefore, mild allergic reactions are frequently encountered in everyday clinical practice. However, even such mild allergic reactions can still lead to serious complications, and therefore, prompt attention, prevention, and treatment are required [2]. In the United States, the Advisory Committee on Immunization Practices (ACIP) recommends an immunization schedule in children to receive a total of 10 vaccines to protect against 16 diseases before the age of two years [3]. According to a recent study, the risk of an allergic reaction post-vaccination is estimated to be 1.31 (95% CI, 0.90–1.84) per million vaccine doses [4]. In this study, age and gender were equal factors, although a slightly higher incidence was observed in females, but mortality was not reported. The US Food and Drug Administration (FDA), announced that the manufacturer’s Special Drug Characteristics (SPC) insert for each of the currently available US vaccines should provide an accurate description of the vaccine’s manufacturing process, and the amount and purpose of each ingredient or excipient substance that is contained in the vaccine [5].

## 2. Pathophysiology of Vaccine-Induced Allergic Reactions

**Figure 1:**
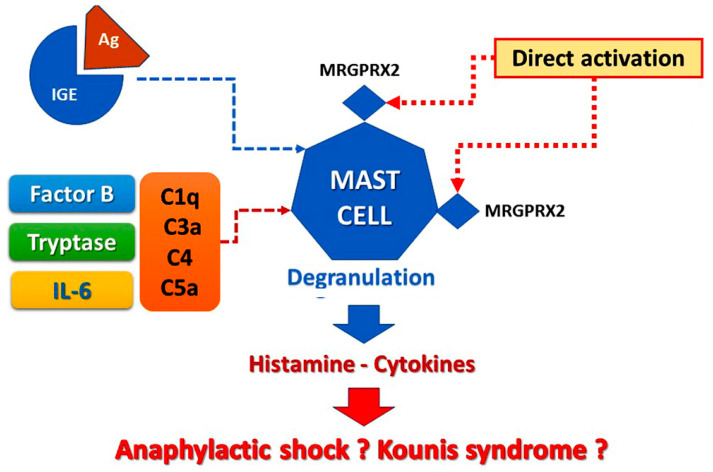
Vaccine-induced allergic reaction.

The allergic reactions caused by vaccines can stem from the following pathophysiologic mechanisms (Figure 1):

a. Reactions via the pathway of mast cell activation and degranulation as IgE/antigen through cross-linking of FcεRI on mast cells [6]. This activation of mast cells via Fcε receptor-1 constitutes the hallmark of classical Gell and Coombs Type I of hypersensitivity reactions [6]. This type of reaction is the most important and well-understood. Especially, type I reactions are mediated by the interaction between IgE antibodies against a particular vaccine component acting as antigen and usually occur within minutes or up to 4 h of exposure to the relevant antigen [2]. This mechanism is confirmed by the specific IgEs detection and the increased levels of serum tryptase [7]. The contained excipients are considered as the most probable cause of IgE-mediated allergic reactions, whereas the vaccine antigen and the residual non human protein should always be regarded as the less probable cause of the allergy, referring to the currently approved COVID-19 vaccine [8].

b. Non-IgE-mediated mast cell degranulation is performed via activation of the complement system that leads to the generation of anaphylatoxins C1q, C3a C4, and C5a and Factor B, which are strong inflammatory stimulators able to induce mast cell activation and degranulation. This complement pathway activation and positive bio-feedback loops involving interleukin-5 (IL-5) and tryptase are much more common than recognized involving patients who develop renal failure or fatal cerebral events [7,9].

c. Life-threatening allergic reactions can be mediated via direct activation of the Mas-related G protein-coupled receptor X2 (MRGPRX2). MRGPRX2 factors that may activate mast cells via non-Fcε receptors are clinically and significantly enhancing our ability to prevent those reactions via MRGPRX2 antagonists’ identification that could further serve as new therapeutic strategies [10]. Notably, in MRGPRX2 activation of mast cells, the specific IgEs may remain undetected, and tryptase levels may be normal even in so serious Kounis syndrome [8].

d. Type IV hypersensitivity or delayed reactions generally initiate 48 h after vaccination demonstrating their peak between 72 and 96 h [2]. They constitute the second most common type of hypersensitivity reactions, are cell-mediated and antibody independent, are derived from overstimulation of T cells and monocytes/macrophages and release of cytokines that cause inflammation, cell death, and tissue damage. Vaccines containing anti-microbial agents and ingredients, such as thimerosal and aluminum, can be followed by delayed reactions [2]. For further understanding of the reactions caused by the current COVID-19 vaccination, it is important to consider all the above mechanisms and pathways.

## 3. Causality of Vaccine Allergy

**Figure 2:**
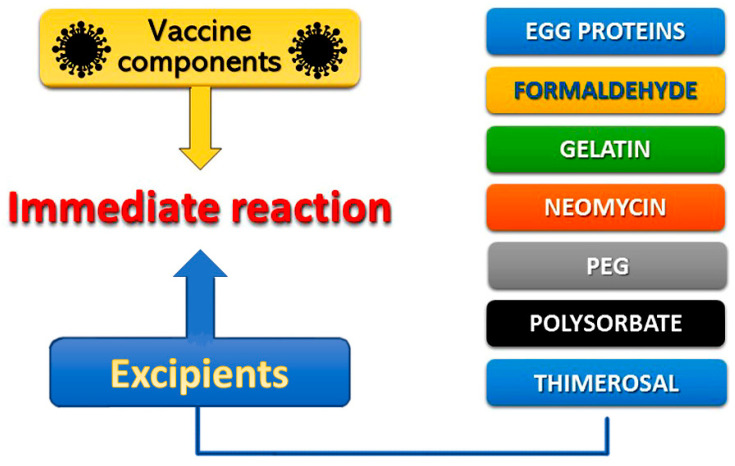
Vaccine compound allergens.

Allergic reactions to vaccines are rarely attributed to the active vaccine itself, as they might be caused to inactive ingredients, such as egg protein, gelatin, formaldehyde, thimerosal, or neomycin, that contribute to specific IgE-mediated immediate reactions (Figure 2). Excipients, according to European Medicines Agency (EMA), are constituents of a pharmaceutical form apart from the active substance [11]. Excipients constitute inert substances that are added to vaccines and other drugs in an effort to improve stability, increase solubility, improve absorption, influence palatability, or create a distinctive appearance. Excipients can cause a variety of allergic clinical manifestations ranging from skin disorders to life-threatening systemic reactions. In particular, necessary excipients added to vaccines to stimulate a stronger immune response, stabilize the potency of the vaccine during transportation and storage, or to prevent contamination by bacteria, can further trigger allergic reactions. Consequently, excipients represent major contributors to the development of specific IgE-mediated and immediate reactions, associated with vaccines [8]. Other excipients, like polyethylene glycol (PEG) and polysorbate, used to improve water-solubility in drugs, may also cause allergic reactions [12]. The variety of excipients contained in various medications is depicted in Table 1. Polysorbate and PEG excipients are components in many vaccines and in injectable medications [13]. Immunoglobulin Μ (IgM) and immunoglobulin G (IgG) can induce complement activation-related pseudo-allergy (CARPA), which constitutes a nonspecific immune response to PEGylated nanoparticle-based medicines [14]. This pathway may be responsible for reactions to medications, such as liposomal doxorubicin and other drugs in clinical trials [15].

On the other hand, allergic reactions to the viral or microbial component itself have been implicated rarely in atopic patients after vaccination with Bordetella pertussis antigens, tetanus, and diphtheria toxoids, or pneumococcus vaccine [16]. Delayed urticaria, angioedema, and nonspecific skin rashes have been reported frequently (5% to 13%) in patients receiving such vaccines [17]. In the above reports, however, detailed and meticulous allergic testing to confirm an immune-mediated allergic reaction was not performed.

## 4. Types of Current COVID-19 Vaccines Worldwide

For the first time, the PEG excipient, also known as macrogol, has been used in the currently used two vaccines against COVID-19 infection:

(1) The Pfizer-BioNTech (Pfizer USA-BioNTech Mainz, Germany) and

(2) Moderna COVID-19 mRNA (Cambridge, Massachusetts, USA) vaccines to stabilize lipid nanoparticles that contain the mRNA (Table 2).

These vaccines do not bear any food, drugs, or latex and their type of PEG differs from the PEG used in other vaccines and healthcare products, due to different molecular weight and further its co-formulation as a stabilizing portion of a liposome [18]. Both vaccines use a lipid nanoparticle (LNP) carrier system to prevent rapid enzymatic degradation of mRNA and facilitate in vivo delivery [19]. The added PEG further stabilizes the lipid-based nanoparticle carrier system by making 2000 lipid conjugates that provide a hydrophilic layer that prolongs half-life [20]. Whereas:

(3) The currently available AstraZeneca vaccine (Weatherall Institute of Molecular Medicine, and Oxford Vaccine Group, UK), and

(4) Johnson & Johnson (New Brunswick, New Jersey, USA) COVID-19 under production vaccine contain the excipient polysorbate 80, also known as Tween 80, and do not contain PEG as Moderna and Pfizer-BioNTech vaccines (Table 3).

Polysorbate, which is structurally similar to PEG, contains polyether domains and presents clinical cross-reactivity with PEG [21]. This excipient may be the allergenic cause for several reports of allergic reactions in patients receiving vaccines, steroids, and chemotherapeutics. Polysorbate constitutes the excipient in 70% of injectable biological agents and monoclonal antibodies [22]. Unfortunately, soon after approval, severe allergic reactions to the mRNA-based vaccines that resolved after treatment were reported [23].

(5) The Sputnik V Gam-COVID-Vaccine [24] is a viral two-vector vaccine based on the recombinant adenovirus types 26 and 5, which are two human adenoviruses—a common cold virus—containing the gene that encodes the spike protein (S) of severe acute respiratory syndrome coronavirus 2 (SARS-CoV-2) to stimulate an immune response. They were biotechnology-derived and contained the SARS-CoV-2 S protein cDNA. The vaccine is given in two doses, with the second dose after 21 days. Apart from Russia, the Sputnik V vaccine is used, so far, by other countries, including Algeria, Argentina, Belarus, Bolivia, Brazil, China, Hungary, India, Iran, Israel, Palestinian territories, Serbia, South Korea, and the United Arab Emirates.

(6) The BBIBP-CorV inactivated vaccine [25] was developed by the Beijing Institute of Biological Products and the Chinese Wuhan Institute of biological products Sinopharm promoted by China’s National Pharmaceutical Sinovac Biotech. Before its double inactivation with β-propiolactone, the virus is cultivated in a qualified Vero cell line for propagation.The supernatant of the infected cells constitutes material on which the inactivation is applied. The vaccine is given in two doses, with the second dose after 21 or 28 days. It has been distributed to several Asian and African countries.

## 5. Mode of Action of Current COVID-19 Vaccines

Coronoviruses belong to the Coronaviridae family and constitute the largest group of host-specific RNA viruses that can infect humans, birds, snakes, bats, and mammals. They are non-segmented, enveloped, single-strand, positive-sense, ribonucleic acid viruses, while their name stems from the crown-like surface projections or the Sun’s corona. The SARS-CoV-2 virus consists of four structural proteins naming the S-spike protein (outer spiky glycoprotein), envelope protein (E), membrane glycoprotein (M), and nucleocapsid protein (N), that can interfere with the host’s immune system, enhancing the attachment and transportation into host cells. The S protein disposesof two domains—the S1 domain that contains the receptor-binding domain mediating the attachment to the receptor cell, and the S2 domain, which facilitates the virus fusion to the host cell.

The entry of SARS-CoV-2 into host cells initiated by the connection of the receptor-binding domain to angiotensin-converting enzyme 2 (ACE-2) receptor, which is the main receptor for SARS-CoV-2 on the host cells surface. S protein is a major target for the vaccines and further viral entry inhibition.

**Figure 3:**
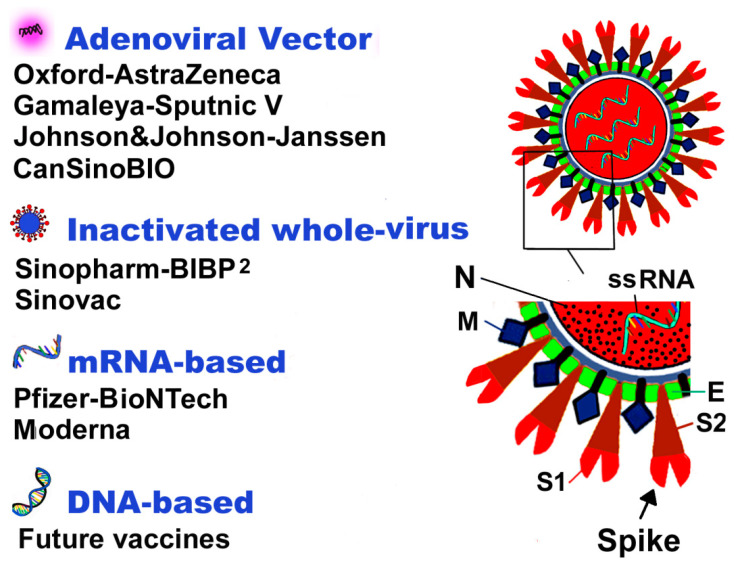
SARS-CoV-2 virus structure and current vaccines. S-spike protein (outer spiky glycoprotein), with the 2 domains (S1 and S2), envelope protein (E), membrane glycoprotein (M), and nucleocapsid protein (N), ssRNA, single-stranded RNA.

The currently available COVID-19 vaccines are based on various platforms and can include (Figure 3):

(1) Traditional inactivated whole-virus vaccines: These vaccines are based on a living virus that has been killed or inactivated, not leading to a clinical disease. This technology is the most traditional and has been exploited by science for vaccine preparation [26]. The inactivated viruses maintain their ability to replicate in vivo with mild or no symptoms. The viruses effectively stimulate the immune system and induce a strong and persistent immune response that prevents infection. Inactivated whole virus includes the entire disease-causing virion (a complete, infective form of a virus outside a host cell, with a core of RNA and a capsid), which is inactivated physically by heat or chemically. The ensuing immune response is directed not only against the S protein, but also against many other SARS-CoV-2 antigens. These vaccines have several advantages, including low production cost, safety, absence of genetic manipulation [27]. The Chinese Wuhan Institute of biological products Sinopharm, the Beijing Institute of biological products Sinopharm, and the Chinese company Sinovac Biotech produce such types of vaccines.

On the other hand, vaccines based on attenuated viral technology have been used previously, but they donot generate a strong immune response unless they use special adjuvants, for example, an aluminum adjuvant. This type of vaccine is not used, so far, for COVID-19. Live attenuated vaccines are found in the market to protect various other diseases, including mumps, rubella, measles, and varicella vaccines

(2) mRNA-based vaccines: In the RNA (Ribonucleic Acid) vaccines or mRNA (messenger RNA) vaccines), short-lived synthetically created molecules of the RNA sequence transfected (infected by transformation) by COVID-19 virus are injected in the individual. The transfected DNA molecules enter into the immunity cells; in this case are taken up in the dendritic cells by phagocytosis of the vaccinated individual to produce an immune response. The infected vaccine’s RNA functions as mRNA inside the immune cells of the vaccinated individual and induce the cells to produce a foreign protein that would normally be produced by a virus. These protein molecules stimulate an adaptive immune responses that can further identify and destroy the corresponding pathogen [28]. mRNA is transported into the human cell in various ways, mostly by lipid microvesicles (liposomes). mRNA vaccines mimic the natural infection of the virus, retaining only a short synthetic viral mRNA that encodes only the required antigen. The procedures to develop the mRNA vaccine include screening of antigens, optimization of sequences, modified nucleotides screening, delivery systems optimization, evaluation safety, and immune response assessment. The mRNA vaccine is far easier to create and design, faster to produce, and stimulates cellular immunity, as well as humoral immunity.

The lipid nanoparticles (LNPs) are the most frequently used vectors for in vivo mRNA vaccine delivery. Indeed, LNPs protect the mRNA against degradation, can be co-delivered with adjuvants, can be synthesized with relative ease in a scalable manner, can be targeted to the desired cell type by surface decoration with ligands, and facilitate endosomal escape. These vaccine preparations have to be kept at −30 to −80 °C.

The Pfizer-BioNTech and Moderna COVID-19 mRNA vaccines are examples of this kind of vaccine.

(3) Adenoviral vector vaccines: In adenoviral vector vaccines, a DNA gene unique to the virus being targeted is added to the viral vector. Such viral vectors are viruses from chimpanzees, gorillas or human adenoviruses. This is done because a preexisting immunity against the virus vector may affect vaccine efficacy. The viral vector is used to shuttle this gene into a human cell. For COVID-19 vaccines, this gene codes the s protein, which is only found on the surface of SARS-CoV-2. Once inside a cell, the viral vector uses this gene and the cell’s mechanism to produce the S protein and display it on the cell’s surface. The virus in which the DNA is inserted may lose its ability to replicate [26,29]. Usually, the virus-based vaccine is administered via intramuscular injection, but there are many interesting projects in administering the vaccine into the nose by inhalation or via other body parts.

Non-replicating vectors are used in Astra Zeneca, Sputnik V Gam-COVID-Vaccine, and Johnson & Jonhson under production vaccine.

## 6. General Therapeutic Considerations

Before administration of any of the currently available COVID-19 vaccines, the individual’s past medical history should be assessed for any previous severe or mild allergic reactions to any cause and especially to the vaccine’s components. The Centers for Disease Control (CDC) and the World Allergy Organization Anaphylaxis Committee [30] have issued clear safety recommendations regarding receiving the vaccine for those with a history of allergic reactions. The Center presented an algorithm to assist with decision-making regarding who can safely receive the COVID-19 vaccine. Individuals with common allergies to several medications, foods, inhalants, insect stings, and latex have the same chance to develop an allergic reaction to the COVID-19 vaccine. Those with a history of a severe allergic reaction due to any cause should be monitored for a 30-min observation period if vaccinated with the COVID-19 vaccine. Anyone with a history of immediate allergic reaction of any severity to any component of mRNA COVID-19 vaccines or to polyethylene glycol or polysorbate should not be vaccinated with the Pfizer-BioNTech or Moderna COVID-19 vaccine.

Allergists opinions and guidance are of paramount importance. It has been found that atopy increases the anaphylaxis risk 2-fold and is not associated to any specific groups of drugs [31]. The questions that physicians should have in mind are related to previous severe or mild allergic reactions and include the followings [13]:

- Mild allergic reactions, such as hives, nasal congestion, rash, scratchy throat, watery or itchy eyes to any injection, or orally and locally given substance.
- Severe allergic reactions, such as angioedema, cardiovascular collapse, including Kounis syndrome, cerebral manifestations, chest tightness, flushing, hives, laryngeal edema, loss of consciousness, low blood pressure (anaphylactic shock), shortness of breath, swelling of mouth, lips, tongue, throat, or wheezing to any oral, local or injectable medication (intravenous, intramuscular, or subcutaneous).
- Severe allergic reaction to a previously injected vaccine.
- Severe allergic reaction to another substance acting as an allergen (e.g., food, venom, or latex
- Severe allergic reaction to PEG or polysorbate.

Patients with a history of severe allergic reactions to PEG or polysorbate should avoid the current vaccination. Patients with a history of severe allergic reactions to other vaccines or injected drugs shouldbe referred to an allergist for further investigation. Patients with a latex allergy should receive the vaccine in an absolutely latex-free environment.

The rest of the patients with a history of allergic reactions, as defined above, should receive the vaccine with a possibility of allergic reaction similar to the general population. Τhe use of antihistamines for prophylactic therapy is a controversial issue, as these medications do not prevent anaphylaxis and could mask cutaneous symptoms leading to a delay in treatment. Some authors, therefore, do not recommend antihistamine pretreatment at this time. Whereas, most antihistamines do not inhibit mast cell degranulation, they can block the histamine receptors. Indeed, in experiments with healthy donors using whole human blood, platelets in plasma and isolated platelets, respectively, the antihistamines inhibited platelet activation-aggregation in the above three experimental groups [32], denoting that these drugs block histamine and adenosine-5 diphosphate receptors situated in platelet surface and can prevent platelet aggregation and thrombosis. Both, H-1 receptor antagonist rupatadine that disposes of anti-PAF and mast cell inhibitory actions, as well as H2-receptor antagonist, famotidine have been reported to be beneficial in COVID-19 [33]. Supplementation with vitamin D3 and liposomal luteolin could also be useful as they can further suppress allergic inflammation and have also been reported to be beneficial in COVID-19 [34].

## 7. Treatment of Severe Systemic Allergic Reactions

The severe systemic allergic reactions are often referred to as anaphylactic reactions representing serious and potentially life-threatening reactions, presented with rapid onset within minutes to hours. In some instances, the evolution of anaphylactic reactions may be delayed, and cardiovascular collapse and/or respiratory failure can occur up to eight hours after symptom initiation. Therefore, the initial and usually milder symptoms should not be ignored [23]. For the Pfizer/BioNTech vaccine, 11.1 cases of allergic reactions (including anaphylaxis) occurred per one million doses, and specifically, 71% of them occurred within 15 min following the first dose [35]. Risk factors, aggravating allergic reactions include previous severe anaphylactic episodes, uncontrolled asthma, mastocytosis, and other mast cell disorders. Additional factors include recent physical exercise, alcohol consumption, non-steroidal anti-inflammatory drugs, or menstruation. Medications like beta-blockers, which are often used in cardiovascular diseases, leave unopposed the action of alpha-adrenergic receptors and can exaggerate the coronary spasm in anaphylaxis. Once anaphylaxis has occurred, epinephrine is the urgent drug of choice, and there is no contraindication to this in the treatment of anaphylaxis because it saves lives. The treatment measures and the order that should be applied are shown in Table 4. The patients, with anaphylactic reactions, who have responded satisfactorily to the first epinephrine injection, could be discharged after 4–8 h following full resolution of the symptoms. However, patients who have received repeated doses of epinephrine need 24-h observation in case of any cardiac arrhythmias and delayed anaphylactic reactions occur [23].

## 8. Patients with Mastocytosis and Mast Cell Disorders

Evidence on whether mast cell activation syndrome or systemic mastocytosis patients may be more prone to COVID-19 infection or develop symptoms post-infection has not been published until now [36]. However, certain conditions affecting the cardiovascular or bronchopulmonary system, and chemotherapy or immunosuppressive drugs may increase the risk of infected patients developing severe COVID-19 symptoms [37]. Therefore, physicians from the medical advisory board of the Mast Cell Disease Society, including the first author (NGK), recommended that patients with mast cell disease should be pre-medicated with an H1 blocker, such as cetirizine 10 mg for adults, one hour prior to the vaccination. However, these recommendations are based on currently available information and may be changed-updated as more clinical data become available. Previous consultation with the pediatrician or allergist is also advised. Some of the physicians also recommend diphenhydramine as an alternative if the individual cannot tolerate cetirizine with the caution that it can cause drowsiness and render some patients, especially the elderly, prone to falls and fractures. In the February 2021 statement of the World Allergy Organization Anaphylaxis Committee was suggested that pretreatment with antihistamines may mask initial symptoms of a reaction [30]. In patients suffering from mastocytosis and mast cell disorders, a discussion with the mast cell disease specialist regarding H1 blockers and the correct dose to be given one hour prior tothe COVID-19 vaccine is advised.

All patients should carry an unexpired Epi-Pen or another form of injectable epinephrine, preferably sulfide-free epinephrine, with them to the vaccine administration site and after vaccination in case of a delayed reaction. Patients with a mast cell disease should receive the vaccine in a healthcare setting where anaphylaxis can be treated, should it occur, and should remain there for 30 min after the vaccine administration. Since the Moderna vaccine is an mRNA vaccine like the Pfizer vaccine, recommendations for use are the same.

## 9. The Second Vaccine Dose in Case of Allergic Reaction to First Dose

Pfizer-BioNTech, Moderna COVID-19 mRNA, and AstraZeneca/Oxford vaccines, as well as the other vaccines, are administered in two doses—whereas, the Johnson & Johnson vaccine would be the first to be given in a single shot, without requiring a booster. The second dose of Pfizer/BioNTech is given 21 days apart, Moderna’s is given 28 days apart, and AstraZeneca/Oxford’s about twelve weeks apart. Until now, there are no data on the safety of the second vaccine after an allergic reaction to the first dose. The authors of this review believe that for patients who developed a convincing severe allergic or anaphylactic reaction to the first dose, skin testing for the causative factor should not be recommended, and avoidance of a second dose of all above vaccines would be a wise decision. However, the patient with type of reaction discussed above should be referred to an allergology center for workup. Skin testing is not always risk-free, and rarely can cause severe anaphylaxis and life-threatening Kounis syndrome [38]. Despite the latter, split dose challenges have been recommended and used in various previously administered vaccines for which the investigators had obtained more allergy experience [39].

## 10. Vasovagal Symptomatology Masquerading as Allergic Reaction

Vasovagal signs and symptoms can occur before and after vaccination, in any individual, but especially in females of all ages and in particular in young people [40]. Whereas, reactions to vaccines are numerically rare, often they can cause substantial fear and anxiety in the general population, contributing to decreased unwillingness to receive a COVID19 vaccine. Such reactions may masquerade as allergic reactions causing confusion in proper diagnosis and treatment (Table 5). It is known that hypotension, syncopal reactions, and other vasovagal reactions are associated with fear of injections, including vaccines, and occasionally are encountered in susceptible individuals. Flushing, tremor, shortness of breath, tachycardia, hyperkinesia, and light-headedness are associated with anxiety and panic, and constitute symptoms masquerading as allergic reactions [13]. Shortness of breath, throat tightness, and inducible laryngeal obstruction, such as vocal cord dysfunction, may also masquerade as anaphylaxis. Frequent episodes of vasovagal symptoms have been described in the human papillomavirus, meningococcal conjugate vaccine, tetanus, diphtheria, and pertussis vaccine.

It is anticipated that physicians dealing with COVID-19 vaccinations will face such reactions, and therefore, should be aware of such situations in order to correctly and safely diagnose patients.

## 11. COVID-19 Vaccination in Pregnancy

According to the CDC and prevention, pregnant women are at increased risk for severe illness from COVID-19, but there is no data on how pregnant women respond to the Pfizer or Moderna vaccines because they were not included in the trials so far. The increased risk of harm from COVID-19 infection in pregnancy, however, highlights the important question of whether pregnant should participate in the development and deployment of COVID-19 vaccines [41]. Experts are still debating if vaccines should, in general, be tested in pregnant women. Inadvertently, replicating vaccines have been performed in and around pregnancy because of unknown pregnancy status. Indeed, major vaccines have not been tested during pregnancy so far because of concerns that both the pregnant person and fetus would be at risk for any complications. The vaccines related to the Ebola virus, yellow fever, and rubella have shown that that contradictory messages around the safety of live vaccines in pregnancy have critical public health costs [42]. On the other hand, restricting the use of replicating vaccines in pregnancy may delay or deny access to the only available protection against deadly diseases. Unvaccinated pregnant people may also slow epidemic control. Finally, uncertainty and worry around the safety of live vaccines may lead to terminations of otherwise desired pregnancies after inadvertent vaccination in pregnancy. Further advice could be obtained from the Advisory Committee on Immunization Practices, which is an independent group created by the CDC, and assist on who and when such a vaccine can be performed, as well as the indications and contraindications related to this specific vaccination [43].

## 12. The FDA Emergency Use Authorization (EUA) for Adverse COVID-19 Events after the First Dose of Pfizer-BioNTech and ModernaVaccines

In the United States of America, during December 14–23, 2020, after administration of 1,893,360 first doses of Pfizer-BioNTech COVID-19 vaccine (1,177,527 in women, 648,327 in men, and 67,506 doses missing sex), 4393 (0.2%) adverse events were reported to Vaccine Adverse Events Reporting System (VAERS). Among these individuals, 175 were identified as possible severe allergic reactions, including anaphylaxis [44,45,46]. The CDC recorded 21 cases that were submitted to and met the Brighton Collaboration case definition criteria for anaphylaxis, corresponding to an estimated rate of 11.1 cases per million doses administered—well above the expected frequency of a severe allergic vaccine reaction, which is 1.31:1 million [4]. Four of these patients (19%) were admitted to hospitals, and three of them were treated in the intensive care unit. Seventeen (81%) were treated in the emergency department, 20 (95%) have been discharged home or had recovered at the time of the report to VAERS. According to VAERS data, deaths from anaphylaxis were not reported. The time of symptom onset was 13 min (range, 2–150 min), 15 patients (71%) had onset within 15 min, 18 (86%) had onset within 30 min. Seventeen (81%) of 21 patients with anaphylaxis had a documented previous history of allergic reactions to drugs, medical products, foods, and insect stings; and 7 (33%) had experienced an episode of anaphylaxis in the past, including one after receipt of rabies vaccine and another after receipt of influenza A (H1N1) vaccine. During the same period, VAERS identified 83 cases of non-anaphylactic allergic reactions, a term used to describe clinically identical allergic reactions that are not immunologically mediated. The clinical diagnosis and management are, however, the same. Commonly reported symptoms in non-anaphylactic allergic reactions included mild respiratory symptoms, itchy and scratchy sensations in the throat, pruritus, and rash.

On 22 January 2021, the Morbidity and Mortality Weekly Report (MMWR) posted that 4,041,396 first doses of Moderna COVID-19 vaccine had been administered in the United States, and 1266 (0.03%) adverse events following vaccination were reported to VAERS. Among these adverse events, 10 were diagnosed with anaphylaxis. This is translated into 2.5 anaphylaxis cases per million that is also above the expected frequency of a severe allergic vaccine reaction of 1.31:1 million, but less than the equivalent Pfizer-BioNTech frequency [47]. Nine of these individuals had a documented history of allergies or allergic reactions, five of whom mentioned a previous history of anaphylaxis.

## 13. Potential Mechanisms of the COVID-19 Induced Allergic Reactions

While prophylaxis means ‘protection’ in Greek, (an)aphylaxis means ‘opposite protection’ or ‘against protection’). Anaphylaxis is a state of life-threatening severe allergic reaction, induced by exposure to certain kinds of antigens [48]. Almost all vaccine components can be considered potential allergic reaction triggers [49]. These include inactivated or killed viruses, and their fragments—such as capsule polysaccharides, conjugating agents, preservatives, stabilizers, antimicrobial agents, and adjuvant and culture media used in the preparation of the vaccine. The sane applies for excipients, used in the manufacturing process, and any inadvertent contaminants introduced during the handling of constituting active immunizing antigens. However, Pfizer-BioNTech and Moderna COVID-19 vaccines do not contain syringe with rubber latex vial stoppers, culture-derived proteins from eggs, yeast, or gelatin used in viral vaccines to stabilize virus viability. All four described pathophysiologic pathways can lead to allergic reactions, namely, mast cell activation and degranulation as IgE/antigen through cross-linking of FcεRI on mast cells, further resulting in complement system induction, activation of MRGPRX2 and overstimulation of T cells and monocytes/macrophages releasing cytokines. Indeed, atopic individuals who have a genetic predisposition to produce significant amounts of antibodies when simultaneously exposed to several allergens can have more symptoms than mono-sensitized individuals [50]. Furthermore, IgE antibodies with different specificities can have additive effects, and small, even sub-threshold IgE numbers can trigger the inflammatory cells to release their mediators and induce allergic reactions. A condition that frequently occurs when the patient is simultaneously exposed to the corresponding antigens [51]. Lipid Nanoparticles used in the Pfizer-BioNTech and Moderna COVID-19 vaccines to protect mRNA against degradation may contain low levels (<2 mol %) of ALC-0159, which further contributes to nanoparticle stabilization by a steric mechanism through its PEG moiety [52]. In the Moderna COVID-19 vaccine, ALC-0159 is replaced with another PEGylated lipid (1,2-dimyristoyl-rac-glycero-3-methoxyPEG2000). Speculations exist on a possible role for these PEGylated lipids in triggering anaphylaxis. Indeed, such PEGylated nanomedicines have been incriminated as inducing anaphylactic reactions [53].

However, anaphylaxis to COVID-19 vaccine components is rarely encountered, and vaccination against COVID-19 is the only promising preventing action for successfully defeating this pandemic.

## 14. Are COVID-19 Vaccines Causing Deaths?

Recently, several cases of death, after vaccination, are being announced through public sites and mass media, which bring chaos in the field and public doubts on the safety of vaccination. A fundamental problem in public health is the distrust in the national vaccine program, and campaigns against vaccines in several countries worldwide are raising concerns on how this pandemic will be defeated. However, purely scientific reports on such events are lacking. No reports of deaths following COVID-19 vaccinations have been published and cited in PubMed. In the 14–23 December 2020 FDA EUA, neither deaths from anaphylaxis nor any deaths associated with COVID-19 vaccination from other etiologies were reported.

However, in Norway, 29 patients died shortly after receiving the vaccine. Physicians have been told to conduct more thorough evaluations of very frail elderly patients in line to receive the Pfizer BioNTech vaccine against COVID-19 [54]. The medical director of the Norwegian Medicines Agency speculated that common adverse reactions of mRNA vaccines, such as fever, nausea, and diarrhea, may have contributed to fatal outcomes in some of the frail patients. Some of these individuals, apparently, experienced anaphylaxis, and concerns have been raised about the cause of such allergy and anaphylaxis [55]. Furthermore, the Paul Ehrlich Institute in Germany is investigating 10 deaths shortly after COVID-19 vaccination [56]. Whereas no deaths have been reported in the United Kingdom, the country’s Medicines and Health care Products Regulatory Agency has expressed concern, and details of all suspected reactions reported, in association with approved COVID-19 vaccines, would be published along with its assessment of the data on a regular basis in the future. The Pfizer and BioNTech are aware of the reported deaths and are working to gather all the relevant information.

We believe that a thorough investigation should be carried out in any unexpected death and allergic reactions associated with current COVID-19 vaccinations. Anaphylactic cardiovascular collapse associated with Kounis syndrome should not be excluded. In severe anaphylaxis associated with sudden death, cutaneous manifestations could be absent and cardiovascular investigations are difficult to obtain. Postmortem tryptase measurement, total, and specific IgEs together with eosinophils and mast cells in the splenic red pulp searching with the implementation of molecular testing at protein or mRNA level could contribute to death cause elucidation and further anaphylactic death confirmation [57].

## 15. Antihistamines, Anti-IgEs, Bronchial Asthma, and COVID-19 Vaccines

The Severe Acute Respiratory Syndrome Coronavirus 2 (SARS-CoV-2) invades the endothelial cells that contain ACE-2 receptors. The ACE-2 metabolizes angiotensin-II to the vasodilatory and anti-inflammatory peptide angiotensin. The SARS-CoV-2 entry in the endothelial cells, especially in the early phases of the infection, interrupts the metabolism of angiotensin II, which results in increased angiotensin-II amounts that induce a significant pro-inflammatory-cytokine release of the cytokine storm [58].

Several H1 receptor antagonists have demonstrated inhibitory properties on the production and expression of interleukins, chemokines, and other cytokines [59]. Specifically, cetirizine decreases interleukin production [60]. Antihistamines, apart from their classical action for blocking H1 and H2 receptors, have been identified, recently, as having powerful antiviral properties and inhibit the entry of certain viruses into the target cell [61,62]. In a recent report, it was shown that treatment with antihistamines, plus azithromycin in selected cases, may treat COVID-19 and prevent progression to severe disease in elderly patients [63].

Furthermore, Ebastine, an H1 histamine inhibitor, and Idelalisib that is a phosphoinositide 3-kinase d (PI3Kd) inhibitor have been proposed as preventive strategies in the airway and allergic diseases. Indeed, these two medications can suppress the release of pro-inflammatory cytokines, such as IL-1b, IL-8, IL-6, TNF-a, and reduce inflammatory reactions and mortality, thus enabling patients to recover faster [64]. It is anticipated that in severely allergic patients, massive doses of antihistamines, as well as Idelalisib, could prevent any severe reactions to the vaccines also.

Immunoglobulin E antibodies are synthesized and released by B lymphocytes as a result of a complex interplay between genes, cytokines, and environmental antigen exposure. They constitute components of a protein network implicated in signaling response to antigens/allergens, and participate in atopic diseases and systemic anaphylaxis [65]. Additionally, IgEs might be increased in acute myocardial infarction, stable and unstable angina, further correlating with plaque destabilization and severity of acute myocardial infarction [66], while elevated IgE levels may be a risk factor for increased cardiovascular mortality [67]. Patients may become IgE-sensitized by previous exposure to antigens [68], therefore atopic patients could be more vulnerable in the second dose of vaccination. Experimental studies indicate that induction of the autoimmune response is dependent on the plasma concentration of IgE before vaccination. A high concentration of IgE has a negative effect on the induction of autoimmunity, most likely by inducing a B-cell tolerance in the host. Vaccinated subjects with very high IgE concentrations thereby respond poorly to the vaccine [69]. Therefore, anti-IgE therapy with monoclonal antibodies will give a better vaccine response and could further prevent allergic reactions.

In February 2021, the United Kingdom Department of Health and Social Care announced that individuals with no-severe asthma are considered by the National Health Service to be at increased risk for COVID-19, but not at risk to die from the coronavirus. The asthmatic patients were characterized as at increased risk of “long covid”—an array of different symptoms lasting weeks to months after the initial infection. This announcement increased confusion about patients with asthma who are going to be prioritized for the COVID-19 vaccination if they repeatedly use systemic steroids or have been hospitalized due to asthma. However, current recommendations of the American Academy of Pediatrics concerning children with asthma suggest that children should be vaccinated in general with inactivated trivalent vaccine and should not receive the live attenuated vaccine [70,71].

## 16. “There Are More Questions Than Answers”

Many vaccines for COVID-19 are going to appear in the market in the near future, including DNA vaccines [28], and our effort as physicians to maintain public confidence and minimize vaccine hesitancy will be crucial. We are aware that any drug, vaccine, medical device, or medical product can induce adverse or allergic reactions. Any uncommon reaction, such as the four cases of Bell’s palsy reported in the Pfizer-BioNTech vaccine trial group, should be thoroughly investigated, and its cause should be determined [68].

Indeed, in the world of COVID-19 and vaccines, there are many questions that remain and need proper answers:

- What is the level of immunity after one shot?
- Which vaccine component is the culprit?
- What happens if the second shot is delayed due to lack of availability?
- How long is the immunity duration?
- How long is the duration of the vaccination?
- When will trials stop in order to have effective vaccines?
- How long is the course of the immunity after vaccination?
- If you already had COVID-19, should you still get vaccinated?
- Are reactions mediated by IgEs?
- Once you get vaccinated, can you transmit the virus or still get sick?
- Is post-vaccination surveillance and documentation a challenge?
- Will we need another vaccination if the virus mutates?
- Are the deaths after COVID-19 due to anaphylactic cardiac collapse and Kounis syndrome?

## 17. Conclusions

Apart from the allergic reactions described in this review, COVID-19 vaccines must be closely monitored in order to detect any additional adverse reactions. Both healthcare professionals and patients should report promptly any unexpected or serious adverse reactions. This is vital because it will aid in their elucidation, prevention, and treatment. All patients with potentially adverse or allergic reactions should be reported through formal processes to CDC and VAERS in the USA or to similar bodies in other countries. Allergists’ expertise in proper diagnosis and treatment of allergic reactions is vital for the screening of high-risk individuals, training of clinic staff conducting vaccinations, and managing patients who experience allergic reactions post-COVID-19 vaccination.

Patients with anaphylaxis should be transferred to the emergency department to receive appropriate medical care. All patients should be instructed to seek immediate medical care if they develop signs or symptoms of an allergic reaction while waiting at the vaccination location. Clinicians play a crucial role in vaccine safety monitoring by being vigilant in recognizing and reporting adverse events.

Careful vaccine-safety surveillance over time, in conjunction with the elucidation of mechanisms of adverse events across different SARS-CoV-2 vaccine platforms, will contribute to the development of a safe vaccine strategy.

## Figures and Tables

**Table 1:** Excipients that can induce allergic reactions, contained in various medications shown in parenthesis.

**Ascorbyl tetraisopalmitate** (topical medications)
**Benzalkonium chloride** (corticosteroids, ophthalmic medications)
**Benzyl alcohol** (parenteral medications, topical medications)
**Carboxymethylcellulose** (corticosteroids, wound dressings)
**Cetostearyl alcohol** (topical medications)
**Chlorocresol** (topical medications)
**Colloidal silica** (NSAID)
**Colophonium** (wound dressings)
**Dioctyl sodium sulfosuccinate** (topical medications)
**Ethylenediaminetetraacetic acid (EDTA)** (topical medications)
**Formaldehyde** (**VACCINES**)
**1,2,6-Hexanetriol** (topical medications)
**Imidazolidinyl urea** (ultrasound gels)
**Isopropyl palmitate** (topical medications)
**Metacresol** (insulin)
**Methyldibromo glutaronitrile** (ultrasound gels)
**Parabens** (topical medications)
**Polyethylene glycol** (**VACCINES**, foods, lubricants, medications, skin creams)
**Polysorbate** (**VACCINES**, anticancer agents, creams, lotions, medications, ointments, vitamin oils)
**Propyl gallate** (topical medications)
**Propylene glycol** (antihistamines, anxiolytics, lubricants, topical medications, ultrasound gels)
**Sodium metabisulfite** (local anesthetics, topical medications)
**Sodium sulfite** (topical medications)
**Sorbitansesquioleate** (topical medications)
**Sunset Yellow** (mineral supplements)
**Thimerosal** (ophthalmic medications)

**Table 2:** (**A**) The Pfizer-BioNTech COVID-19 Vaccine excipients-ingredients. (**B**) The Moderna COVID-19 Vaccine excipients-ingredients.

**A. The Pfizer-BioNTech COVID-19 Vaccine Excipients-Ingredients**
1. mRNA, nucleoside-modified messenger RNA (modRNA) encoding the viral spike (S) glycoprotein of SARS-CoV-2and constitutes the active ingredient
2. Electrolytes potassium chloride, monobasic potassium phosphate, sodium chloride, dibasic sodiumphosphate dihydrate,
3. Lipids ((4-hydroxybutyl)azanediyl)bis(hexane-6,1-diyl)bis(2-hexyldecanoate), [(polyethyleneglycol [PEG])-2000]-N,N-ditetradecylacetamide, 1,2-distearoyl-sn-glycero-3-phosphocholine, and 0.2 mgcholesterol)
4. polyethyleneglycol
5. Sugar (sucrose)
6. Saline (Sodium Chloride) acting as adiluent, added to the vaccine forinjection
**B. The Moderna COVID-19 Vaccine Excipients-Ingredients**
1. Messenger ribonucleic acid (mRNA) as an active ingredient
2. Acetic acid
3. Lipids (SM-102, polyethylene glycol [PEG] 2000 dimyristoyl glycerol [DMG], cholesterol, and 1,2-distearoyl-sn-glycero-3-phosphocholine [DSPC])
4. polyethylene glycol
5. Sodium acetate
6. Sugar (sucrose)
7. Tromethamine (treat or prevent acidosis)
8. Tromethamine hydrochloride

**Table 3:** The AstraZeneca COVID-19 Vaccine excipients-ingredients.

1. Recombinant, replication-deficient chimpanzee adenovirus vector encoding the SARS CoV 2 Spike glycoprotein. Produced in genetically modified human embryonic kidney (HEK) 293 cells
2. Histidine
3. L-histidine hydrochloride monohydrat
4. Magnesium chloride hexahydrate
5. Polysorbate 80
6. Ethanol
7. Sugar (sucrose)
8. Sodium chloride
9. Disodium edetate dihydrate
10. Water for injections

**Table 4:** The decalogue of treatment measures should be applied in the following order.

1. Patient in recline position with legs up and administer intramuscular epinephrine
2. Intravenous line for volume replacement with intravenous 0.9% NaCl
3. Airways clearing, vital signs checking, oxygen via facial mask at least 10 L/minute administration
4. 2–3 L of intravenous 0.9% NaCl in 10–20 min if hypotension and rapid volume loss
5. Repeat intramuscular epinephrine if no improvement within 5–10 min and call emergency assistance
6. Short-acting beta-agonists [salbutamol) puffs via large volume spacer for severe dyspnea/wheezing
7. Glucagon in patients on beta-blockers who are unresponsive to epinephrine
8. Nebulized epinephrine and nebulized short-acting beta-agonists in cases of signs of severe upper airway obstruction (laryngeal/uvula/tongue edema)
9. Oral or intravenous glucocorticoids and oral or intravenous antihistamines
10. Measuring mast cell tryptase2–3 h after the beginning of the reaction to confirm anaphylaxis

**Table 5:** Vasovagal reactions.

**Symptoms and Signs**
1. Precede or occur after a few seconds to a few minutes after the injection
2. Fainting sensation, light-headedness dizziness, blurry vision, loss of consciousness in some cases
3. Grey or pale skin appearance
4. Vertigo, weakness
5. Blurred, faded, narrowing, or tunnel vision
6. Paresthesias
7. Feeling of warmth
8. Slow breathing, with a few seconds of apnea in some cases
9. Regular butslow and weak pulse
10. Cold, sweaty, clammy skin
11. Transient hypotension
12. Uncontrollable yawning, nausea, vomiting nausea, vomiting, epigastric discomfort, abdominal pains, diarrhea
**Management**
Continuous reassurance, well-ventilated room, cold and damp cloth on forehead and face, recumbent position with elevated legs above head or have the patientput their head between their knees
**Prevention**
Vaccination should be performed in a sitting position. Before vaccinating, ask if the patient tends to faint; if so, ask the patient to lie down

## Data Availability

Not applicable.

## Abbreviations

ACE-2	Angiotensin-Converting Enzyme 2
ACIP	Advisory Committee on Immunization Practices
CARPA	Complement Activation-related Pseudo-Allergy
CDC	Centers for Disease Control
COVID-19	Coronavirus Disease 2019
EUA	Emergency Use Authorization
EMA	European Medicines Agency
FDA	Food and Drug Administration
IgE	Immunoglobulin E
IgG	Immunoglobulin G
IgM	Immunoglobulin M
LNP	Lipid Nano Particles
MRGPRx2	Mas-related G Protein-Coupled Receptor X2
MMWR	Morbidity and Mortality Weekly Report
PAF	Platelet Activating Factor
PEG	Polyethylene Glycol
RNA	Ribonucleic Acid
mRNA	messenger Ribonucleic Acid
SARS-CoV-2	Severe Acute Respiratory Syndrome Coronavirus 2
VAERS	Vaccine Adverse Events Reporting System (VAERS).
